# Different Expressions and Methylation Patterns of cGAS and STING in Cervical Cancer

**DOI:** 10.1155/ogi/2210380

**Published:** 2026-01-30

**Authors:** Ruimin Wang, Shuling Liu, Rui Wang, Quanquan Guo, Xiaoyuan Lu

**Affiliations:** ^1^ Department of Obstetrics and Gynecology, Suqian Affiliated Hospital of Xuzhou Medical University, No. 138 Huanghe Road, Suqian City, 223806, China; ^2^ Department of Obstetrics and Gynecology, the Affiliated Hospital of Xuzhou Medical University, Xuzhou, 221002, China, xzmc.edu.cn; ^3^ Department of Oncology, Pudong Gongli Hospital, Shanghai University of Medicine & Health Sciences, Shanghai, China, sumhs.edu.cn; ^4^ Department of Oncology, Suqian Affiliated Hospital of Xuzhou Medical University, No. 138 Huanghe Road, Suqian City, 223806, China; ^5^ Department of Hematology, the Second Affiliated Hospital of Soochow University, No. 1055 San Xiang Road, Su Zhou City, 215004, China, suda.edu.cn

**Keywords:** cervical tumor, cGAS, methylation, STING, TCGA

## Abstract

**Background:**

The cGAS–STING pathway has established itself as a critical innate immune pathway that has the ability to significantly affect tumor initiation and progression. The expression, methylation, immunological functions, and prognostic importance of cGAS–STING pathway‐related genes in cervical squamous cancer (CESC) patients have not yet been thoroughly elucidated.

**Methods:**

First, we explored the expression of cGAS and STING in cervical carcinoma samples from TCGA by comparing the mRNA and protein levels of cGAS and STING in both TCGA cervical tumor patient samples and cervical tumor cell lines. Second, we examined the CD4^+^T and CD8^+^T cell infiltration in STING high and low samples and made Kaplan–Meier prognosis analysis of STING protein expression. Third, to verify the findings in TCGA public datasets, we retrospectively selected 40 cervical squamous carcinoma patients and 10 normal cervical tissues and evaluated cGAS and STING protein expression using immunohistochemistry (IHC). All patients have detailed clinical information, which includes age, FIGO stage, menstruation status, follow‐up time, histology, tumor diameter, and serum tumor markers.

**Results:**

In both cervical tumor patient samples and cell lines, we observed that cGAS is increased, whereas STING is decreased in tumors, which leads to decreased CD4^+^T and CD8^+^T cell infiltration and poor prognosis. Furthermore, the cGAS mRNA transcript showed a gradual increase and STING mRNA showed a decrease according to the tumor stage, tumor grade, metastasis status, and histology types. We confirmed the expression of cGAS and STING proteins in clinical cervical tumor samples using IHC. Mechanically, cGAS and STING showed different DNA methylation patterns, which might contribute to the differences in cGAS and STING mRNA and protein levels.

**Conclusions:**

Our work identified different expressions and methylation patterns of cGAS and STING in cervical cancer and their correlation with immune T cell infiltration and prognosis. More mechanistic study is needed to understand the cGAS–STING pathway in cervical squamous tumor.

**Highlights**

• cGAS is upregulated and STING is downregulated in cervical squamous cancer.
• The cGAS DNA promoter is hypomethylated, and the STING DNA promoter is hypermethylated.
• Low STING correlates with effector T cell infiltration and poor prognosis.

**Significance**

• STING functions as a crucial innate immune sensor for cytosolic DNA, triggering vital antiviral and antitumoral reactions. The findings of this study demonstrate that STING expression is downregulated in both cervical tumor cell lines and patients’ tumor samples, with high tumoral STING expression correlating with T cell infiltration and good relapse‐free survival (RFS) and overall survival (OS). On the contrary, cGAS expression is upregulated in both cervical tumor cell lines and patient tumor samples, with high tumoral cGAS expression correlating with a low OS and RFS. This previously unreported divergent trend of cGAS and STING presents a novel phenomenon, which needs future mechanistic study.

## 1. Introduction

Cervical cancer continues to be the second most prevalent cause of gynecologic cancer‐related mortality among women worldwide, and it is a global health crisis [1]. Each year, there are approximately 266,000 fatalities and 529,000 newly diagnosed cervical cancer patients globally [2]. Among all factors, chronic infections caused by high‐risk human papillomavirus (HPV) account for over 95% of cervical cancer cases [3, 4]. In cervical cancer patients, a poor prognosis is the result of local invasion and distal lymphatic metastasis. Prophylactic vaccines against carcinogenic HPV varieties are available; however, their efficacy has been restricted posterior to HPV infection [5]. Women who have already contracted HPV are predisposed to an increased risk of getting cervical cancer in the future [6], as it is contingent upon the immune system’s functionality. During this period, cGAS–STING, which are core components of innate immunity, play a significant role in adaptive immunity’s priming, recruitment, and functionality [7, 8]. Consequently, in cervical carcinoma, the cGAS–STING signaling pathway needs further illumination by new information, which aids in the comprehension of the disease’s mechanisms.

Since the identification of cGAS–STING recognizing endogenous DNA released from dead cancer cells and triggering Type I interferon (IFN) and antitumor T cell responses, efforts have been made to understand and therapeutically target the STING pathway in cancer [9]. STING is activated via the direct interaction with cytosolic DNA or 2′3′‐cGAMP, which is synthesized by cyclic GMP–AMP synthase (cGAS) upon detecting cytosolic double‐stranded DNA. Upon activation, STING undergoes a conformational shift that allows it to move from the endoplasmic reticulum (ER) to the Golgi through intermediary compartments between the ER and Golgi [10]. Tank‐binding kinase 1 (TBK1) and IκB kinase (IKK) are recruited and activated during this process. In order to facilitate the nuclear entry of these transcription factors and the synthesis of Type I IFNs, the STING complex recruits and stimulates the expression of IFN Regulatory Factor 3 (IRF3) and NF‐κBat perinuclear regions [11].

This inflammation promotes the activation of spontaneous antitumor T cell responses. DNA or CDNs from dying cancer cells can induce antigen‐presentation activity, Type I IFN signals, costimulatory ligand expression on antigen‐presenting cells (APCs), and chemokines (e.g., CXCL9 and CXCL10) that promote T cell migration to the tumor site in a manner that aligns with STING signaling cascade [12]. Despite these discoveries, there are reports that cGAS and STING have some independent functions. For example, cGAS is reported to inhibit homologous recombination and promote tumorigenesis [13], while cGAS prevents hepatocellular carcinoma cell lines from undergoing ferroptosis and promotes tumor growth [14]. However, it has been reported that high STING levels can impair the functions of NK cells and induce immune inhibitory B cells [15].

In this study, we explore the expression of cGAS and STING in cervical carcinoma samples from TCGA and clinically available patients, categorized by TNM staging and tumor grade. The gene expression profiles and clinicopathological characteristics were acquired from the datasets of the Cancer Genome Atlas (TCGA) [16]and UALCAN datasets [17, 18]. The Kaplan–Meier Plotter and TIMER 2.0 [20] dataset tools were used to perform prognostic evaluation of genes downstream of STING (TBK1, IRF3, CXCL9, CCL5, and CXCL10) and the immunological CD4^+^T and CD8^+^T infiltration profile. The cervical tissues were obtained after hysterectomy, and immunohistochemistry (IHC) was conducted to quantify the protein expressions of cGAS and STING. We observed an evolving rise in cGAS expression, accompanied by a gradual decline in STING expression. This previously unreported divergent trend of cGAS and STING presents a novel phenomenon that has never been reported.

## 2. Materials and Methods

### 2.1. Patients’ Cohort Features

All patients who met the following criteria were eligible to participate in the study: (a) newly diagnosed cervical cancer that was pathologically confirmed; (b) had surgery or a pathological examination performed at Suqian Affiliated Hospital of Xuzhou Medical University from January 2021 to December 2023; (c) tumor staging of CIN1–II B based on the staging system of the 2009 International Federation of Gynecology and Obstetrics; and (d) had no history of radiation or cytotoxic agent exposure. The exclusion criteria were patients who had viral and bacterial infections, autoimmune disorders, and any significant surgical procedures performed 3 months before.

### 2.2. Sample Collection and IHC Staining

Selected cervical tumor samples were obtained from patients who received surgical procedures or pathological examinations. In order to facilitate subsequent IHC labeling, all specimens were immersed in a 4% paraformaldehyde solution. The study received approval from the Ethics Committee and Review Board of the Suqian Affiliated Hospital of Xuzhou Medical University (Approval Number 2023‐S075). Ethical approval for this study was granted by the Institute Ethical Committee (IEC) prior to its initiation. Written informed consent was obtained from all participating women.

A detailed description of the IHC staining processes was described earlier [21]. In summary, the cervical tissue that had been fixed with formalin was sectioned at a thickness of 5 μm. Next, these sections underwent deparaffinization and rehydration using a series of graded ethanol solutions. Finally, the samples were washed with a Tris‐buffered saline solution comprising 20 mmol/L of Tris–HCl and 150 mmol/L of NaCl and maintained at a pH score of 7.6. The antigen retrieval process involved immersing the slides in a sodium citrate buffer with a concentration of 10 mmol/L and a pH of 6.0 for a duration of 15 min. First, the primary antibodies used were anti‐cGAS (rabbit antihuman C6orf150, classified as cell signaling, Cat. No. 83623) and anti‐STING (rabbit antihuman TMEM173, classified as cell signaling, Cat. No. 13647), which were blocked using 10% FBS buffer.

### 2.3. IHC Scoring Criteria

Interpretation and analysis of IHC results are as follows: Two pathologists independently examined the radiographs without knowledge of the patient’s clinical history. Each slide was investigated individually using a light microscope. When the results of two pathologists’ reviews are incongruent, the conclusion of the review is reached through mutual consultation between the two pathologists.

The following criteria were used to interpret the cGAS and STING staining results: The intensity and proportion of positive cells were used to evaluate the immunostaining for cGAS and STING. The staining intensity scores were as follows: 0 (Negative), 1 (Mild Positive), 2 (Medium Positive), and 3 (Strong Positive). The following four kinds of scores were calculated based on the proportion of cGAS (or STING)‐positive cells: 0%–10% was 1, 11%–50% was 2, 51%–80% was 3, and 81%–100% was 4. As indicated previously, the final cGAS (or STING) staining score was calculated by multiplying the intensity score by the percentage score. Positive results were defined as > 10% of cells with dark brown nuclei staining, and negative results were defined as < 10% of cells with staining. We determined the cutoff point for cGAS (or STING) IHC scores using the X‐tile software (Rimm Lab at Yale University, http://www.tissuearray.org/rimmlab).

### 2.4. Sample Size

A sample size calculation was performed using the sensitivity and specificity values (71.7% and 90.9%, respectively). To achieve a study power of 85% and a 15% margin of precision at a 5% significance level, approximately 50 subjects were deemed necessary.

### 2.5. Transcript and Protein Expression and Methylation Analysis

cGAS and STING expressions were analyzed in 22 human cancer types and normal tissues using data from the Human Protein Atlas accessible at https://ualcan.path.uab.edu/analysis.html [17, 18]. The expression of cGAS and STING in various human cervical cancer cell lines was analyzed using experimental data from the Human Protein Atlas Cell Line RNA database accessible at https://www.proteinatlas.org/ENSG00000184584-STING1/cellline [16]. An analysis of CD4^+^T and CD8 T cell infiltration associated with STING was performed with TIMER 2.0 available at http://timer.cistrome.org [20]. The analysis of cGAS and STING protein expressions in cervical squamous carcinoma (CESC) and normal cervical tissues was conducted using the Human Protein Atlas database available on the UALCAN at http://ualcan.path.uab.edu/analysis-prot.html [17, 18]. Using the STING identity “TMEM173,” the expression of STING was obtained from the Human Protein Atlas, TIMER 2.0, and UALCAN databases. STING staining was quantified with the HPA038116 antibody.

### 2.6. Analysis of Prognosis

Kaplan–Meier survival analysis was performed to assess the correlation between cGAS and STING expression, and the OS and RFS of CESC using the Kaplan–Meier Plotter program (https://kmplot.com/analysis/index.php?p=service [19]). The “Auto select best cutoff” option was used to determine the ideal cutoff point for the cGAS and STING expression.

### 2.7. Statistical Analysis

The statistical analyses were performed using the GraphPad Prism program v.8, and all data were reported as mean ± S.D. The statistical analysis of various groups was conducted using either a one‐way ANOVA or a two‐way ANOVA with the Dunnett multiple comparisons test, as necessary. The Kaplan–Meier method was employed to generate survival curves, which were subsequently analyzed using the log‐rank (Mantel–Cox) test. ^∗∗∗^, *p* < 0.001; ^∗∗^, *p* < 0.01; ^∗^, *p* < 0.05 for all analyses.

## 3. Results

### 3.1. cGAS mRNA Is Upregulated in Cervical Squamous Tumor Samples

We made a comprehensive investigation of the mRNA in pan cancer transcript data of *cGAS(c6orf150)* across 22 cancer types from the TCGA database (https://ualcan.path.uab.edu/analysis.html)accessible) through the UALCAN server (https://ualcan.path.uab.edu/index.html [17]) (Figure 1(a)). We found that *cGAS* mRNA is upregulated in most cancer types, compared with the normal counterparts. Cervical squamous tumors exhibited a much significantly higher level of *cGAS* mRNA, compared to the normal tissues (Figure 1(a)), indicating that elevated cGAS may have a substantial impact on tumorigenesis in CESC. Subsequently, we assessed the correlation between clinicopathological characteristics and cGAS expression in TCGA CESC patients. We next used the TCGA database to analyze and compare *cGAS mRNA* with multiple clinic factors. We find that *cGAS* mRNA is higher in CESC tumors (https://ualcan.path.uab.edu/cgi-bin/TCGAExResultNew2.pl?genenamC6ORF150ctypeCESC [18]), compared with the normal tissues (Figure 1(b)). *cGAS* mRNA showed a gradual increase according to TNM Stages I, II, III, and IV (Figure 1(c)). Additionally, CESC with nodal metastasis shows a bit lower level of *cGAS* mRNA (Figure 1(d)). In different tumor histology types, *cGAS* mRNA showed a variety of levels, with adenosquamous histology type being the lowest *cGAS* and endometroid the highest *cGAS* (Figure 1(e)). Nevertheless, all histology types exhibited a significantly elevated level of cGAS mRNA, compared with the normal ones. The prognostic value of cGAS in CESC was subsequently evaluated using an online tool, Kaplan–Meier Plotter (https://kmplot.com/analysis/index.phppservice [19]). The results indicated that patients with high cGAS expression had a worse OS and RFS than those with low cGAS expression in CESC (Figures 1(f), 1(g)).

Figure 1cGAS mRNA is upregulated in cervical squamous tumor samples. (a) Pan cancer analysis of the mRNA transcript data of *cGAS (c6orf150)* across 22 cancer types from the TCGA database. *cGAS* mRNA is upregulated in most cancer types, compared with the normal counterparts. Cervical squamous tumors exhibited a much significantly higher level of *cGAS* mRNA. (b) *cGAS* mRNA showed an increase in the tumor. (c) *cGAS* mRNA exhibited a gradual increase according to TNM Stages I, II, III, and IV. (d) *cGAS* mRNA is higher in tumors with N1 nodal metastasis. (e) *cGAS* mRNA differed in tumor histology, with adenosquamous histology type being the lowest *cGAS* and endometroid the highest *cGAS*. (f) The relationship between the cGAS expression and patient outcome was investigated using the KM Plotter database. High cGAS predicts poor OS and RFS. (g) Patient outcomes in the TCGA cervical squamous tumor dataset. Each value represents mean ± S.D. We compared multiple groups using one‐way ANOVA with Dunnett’s post hoc test for multiple comparisons. ^∗∗∗^, *p* < 0.001; ^∗∗^, *p* < 0.01; ^∗^, *p* < 0.05.

**Figure:**
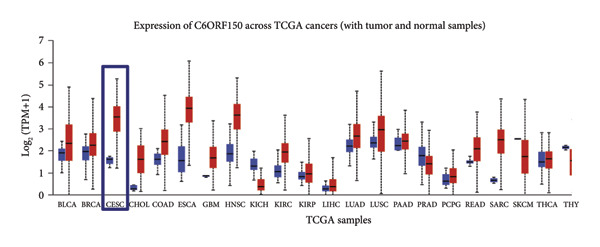
(a)

**Figure:**
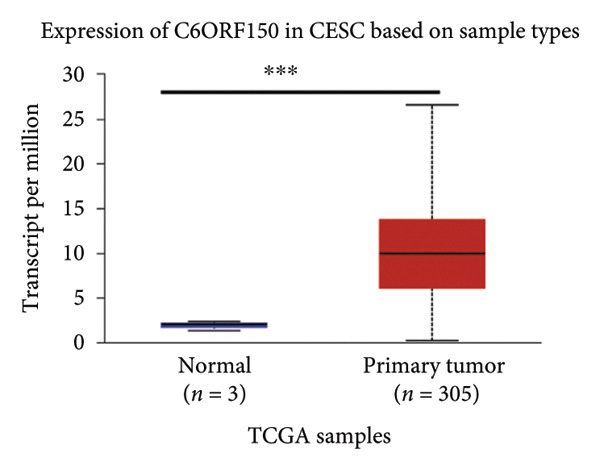
(b)

**Figure:**
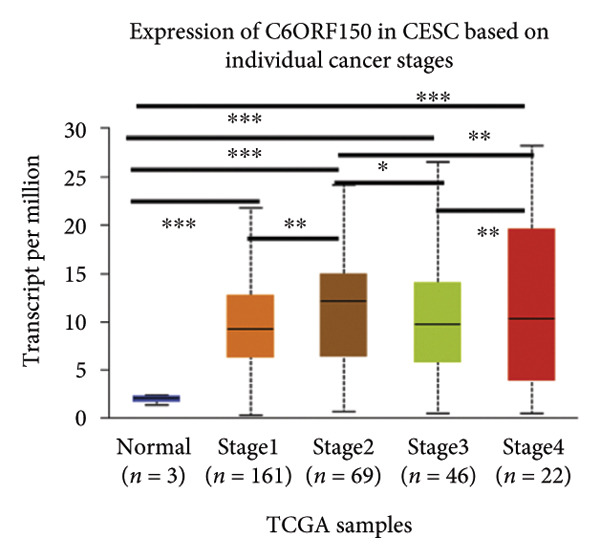
(c)

**Figure:**
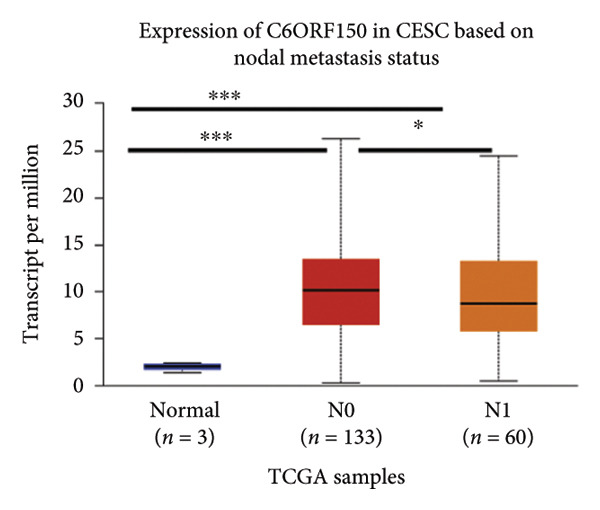
(d)

**Figure:**
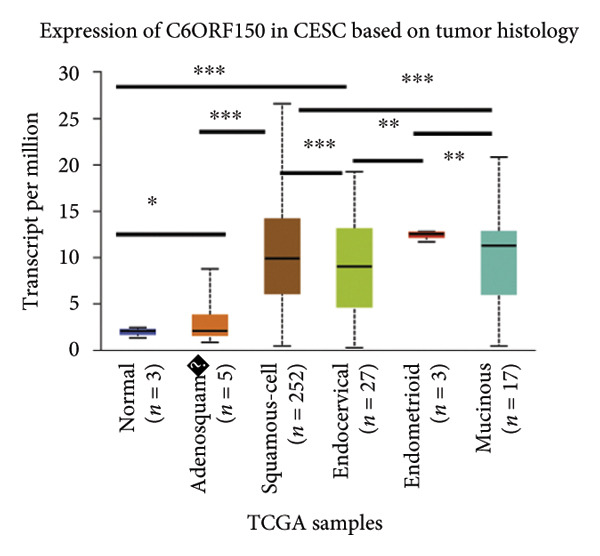
(e)

**Figure:**
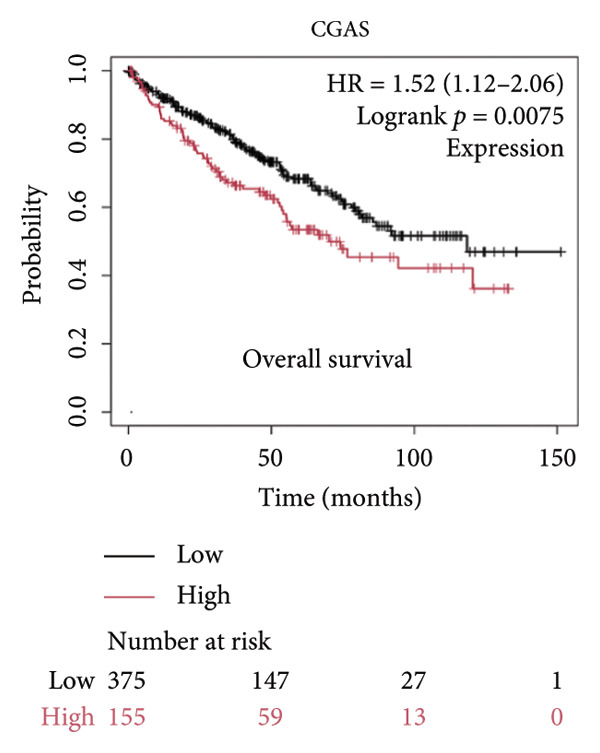
(f)

**Figure:**
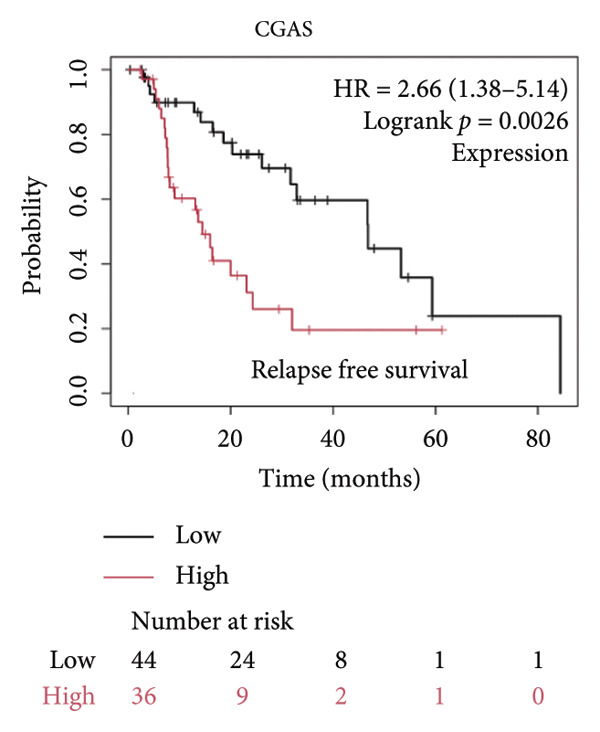
(g)

### 3.2. STING mRNA is Downregulated in Cervical Squamous Tumor Samples

Next, we generated the mRNA transcript data of STING (TMEM173) across 22 cancer types from the TCGA database to gain a better understanding of the role of STING in malignancies (https://ualcan.path.uab.edu/analysis.html [18]) accessible through the UALCAN server (https://ualcan.path.uab.edu/index.html [17]). We discovered that the majority of cancer types exhibit a downregulation of STING mRNA in comparison with their normal counterparts (Figure 2(a)). It is important to note that cervical squamous tumors exhibited a significantly reduced level of STING mRNA in comparison with the normal tissues (Figure 2(a)). This suggests that low/negative STING may play a significant role in the tumorigenesis of CESC. Furthermore, we proceeded to evaluate the association between clinicopathological features and STING expression in patients with TCGA CESC. Again, we used the TCGA database (https://ualcan.path.uab.edu/analysis.html [18]) for analysis. The results indicated that the expression of STING mRNA is further reduced in distant metastatic tumors in comparison with the nonmetastatic tumors (Figure 2(b)). STING mRNA showed a gradual decrease according to TNM Stages I, II, III, and IV (Figure 2(c)). CESC with nodal metastasis showed an even lower level of STING mRNA (Figure 2(d)), which might suggest that patients with limited STING expression exhibited a higher frequency of lymph node metastasis. STING mRNA levels varied greatly among various tumor histology types, with adenosquamous histology exhibiting the lowest STING and mucinous the highest (Figure 2(e)). Nevertheless, the expression level of STING mRNA was markedly decreased in all histology types, compared with the normal histology. STING’s prognostic value in CESC was subsequently assessed using an online tool, Kaplan–Meier Plotter (https://kmplot.com/analysis/index.php?pservice [19]). Patients exhibiting low STING expression in the CESC exhibited poorer OS and RFS compared with those with high STING expression (Figures 2(f), 2(g)).

Figure 2STING mRNA is downregulated in cervical squamous tumor samples. (a) Pan cancer analysis of the mRNA transcript data of *STING (Tmem173)* across 22 cancer types from the TCGA database. *STING* mRNA is upregulated in most cancer types, compared with the normal counterparts. Cervical squamous tumors exhibited a much significantly higher level of STING mRNA. (b) *STING* mRNA showed an increase in the tumor. (c) *STING* mRNA exhibited a gradual increase according to TNM Stages I, II, III, and IV. (d) *STING* mRNA is higher in tumors with nodal metastasis. (e) *STING* mRNA differed in tumor histology, with adenosquamous histology type being the lowest STING and endometroid the highest *STIN*G. (f) The relationship between the STING expression and patient outcome was investigated using the KM Plotter database. High STING predicts poor OS and RFS. (g) Patient outcomes in the TCGA cervical squamous tumor dataset. Each value represents mean ± S.D. We compared multiple groups using one‐way ANOVA with Dunnett’s post hoc test for multiple comparisons. ^∗∗∗^, *p* < 0.001; ^∗∗^, *p* < 0.01; ^∗^, *p* < 0.05.

**Figure:**
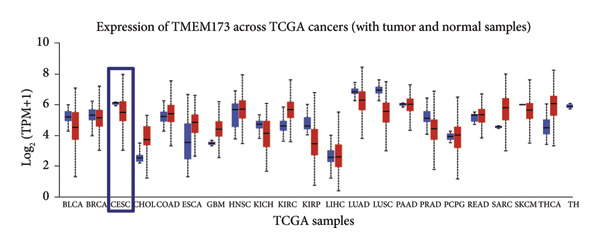
(a)

**Figure:**
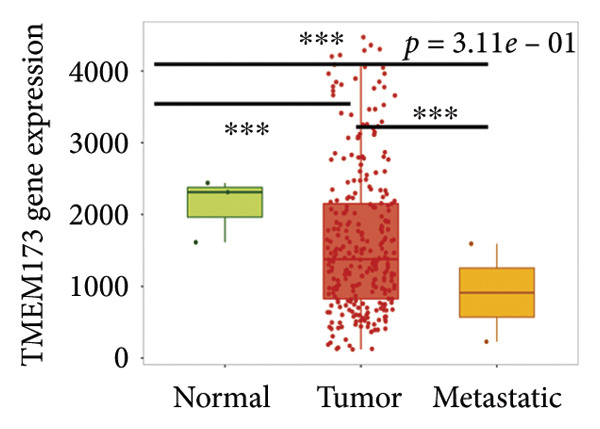
(b)

**Figure:**
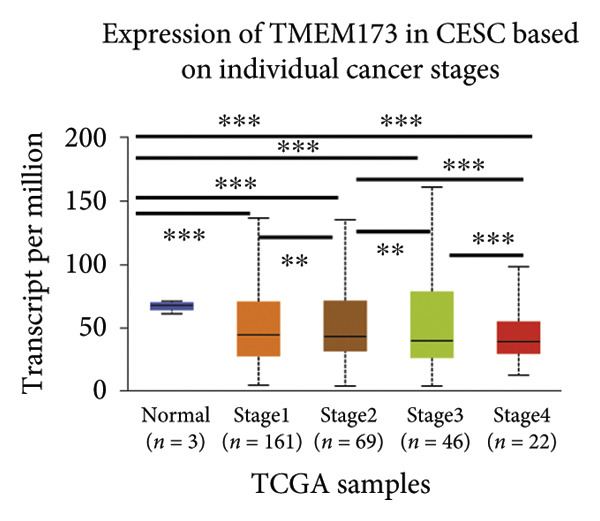
(c)

**Figure:**
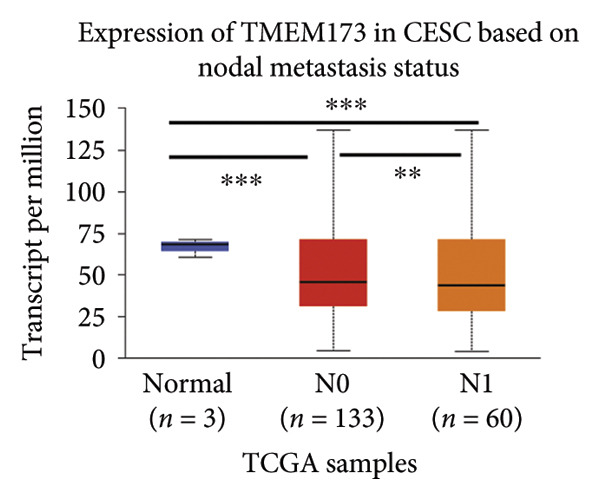
(d)

**Figure:**
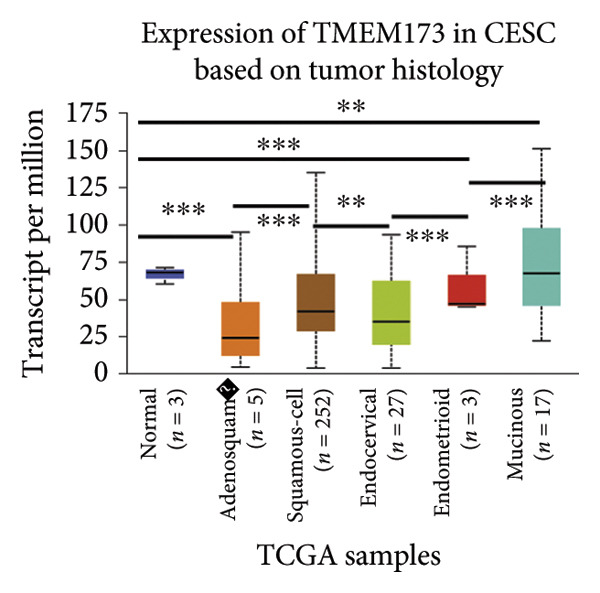
(e)

**Figure:**
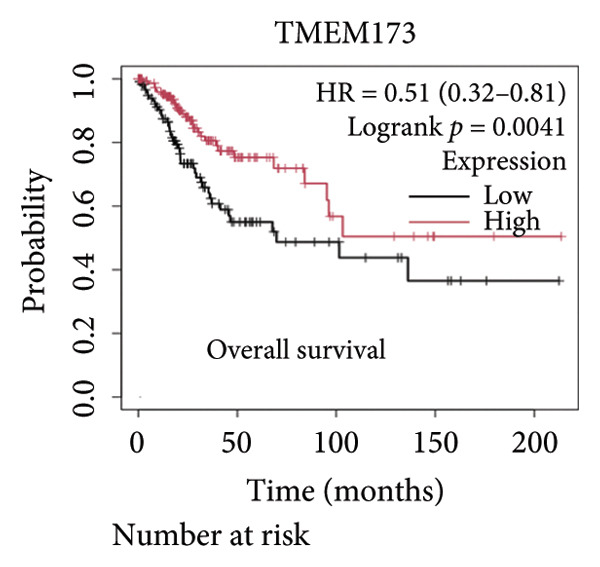
(f)

**Figure:**
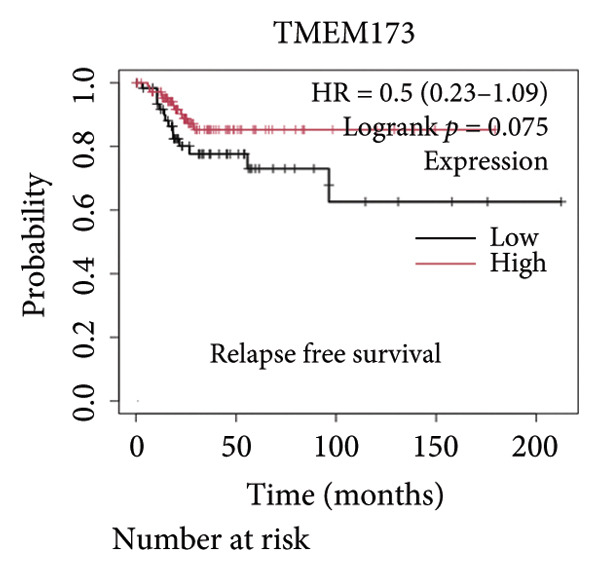
(g)

### 3.3. Tumor Samples and Cell Lines Both Showed cGAS High and STING Low Patterns

We next performed a comparative investigation of cGAS and STING protein expression in Human Protein Atlas pathology for cervical squamous tumors (https://www.proteinatlas.org/ENSG00000184584-STING1/pathology/cervicalcancerihc [16]). Additionally, we compared the mRNA transcripts of cGAS and STING in several cell lines (https://www.proteinatlas.org/ENSG00000184584-STING1/cellline). CESC showed a significantly higher cGAS protein expression (Figures 3(a), 3(b)) and much lower/negative STING protein expression (Figures 3(c), 3(d)). Fifteen out of the 23 CESC patients included in the Human Protein Atlas database did not show measurable levels of STING protein (https://www.proteinatlas.org/ENSG00000184584-STING1/pathology/cervicalcancerihc [16]). On the contrary, the cGAS protein was moderately to strongly positive in all 23 CESC patients (https://www.proteinatlas.org/ENSG00000164430-CGAS/pathology/cervicalcancerihc [16]). It is evident from the cell line categories’ bar chart that both cGAS and STING exhibited low specificity for cancer cell lines, as evidenced by the entire cell lines’ bar chart (Figures 3(e), 3(h)). Nevertheless, the cGAS mRNA level in the majority of cancer cell line types is substantially higher than that of the noncancerous type (labeled by the two arrows). Adrenocortical cancer exhibited the highest cGAS transcript, while cervical cancer also exhibited a significantly higher cGAS level in comparison to the noncancerous (the noncancerous level is labeled at the level) (Figure 3(e)). In order to examine each cancer cell line in greater detail, we demonstrated that the average cGAS transcript level in eight cervical cancer cell lines was significantly higher than that of the 63 noncancerous cell lines (Figures 3(f), 3(g)). In contrast, the average STING transcript level in eight cervical cancer cell lines was significantly lower than that of the 63 noncancerous cell lines (Figures 3(i), 3(j)). Collectively, these data suggest that cGAS increases in CESC, while STING is reduced at both the mRNA and protein levels.

Figure 3Tumor samples and cell lines both showed cGAS high and STING low patterns. (a, c) Representative immunohistochemistry images of cGAS and STING staining in CESC were obtained from Human Protein Atlas pathology. (b, d) Bar chart to show cGAS and STING IHC pathology reading score from 0 to 10. (e) Bar chart to show cGAS mRNA transcript in multiple tumor cell lines, with noncancerous cell lines as the baseline control. Most tumor cell lines had higher cGAS than noncancerous cell lines (the average value of mRNA transcript from multiple cell lines). (f) A detailed bar chart to show the cGAS mRNA transcript in eight CESC cell lines and 63 noncancerous cell lines. (g) On average, CESC cell lines showed significantly higher cGAS mRNA than noncancerous cell lines. (h) Bar chart to show the STING mRNA transcript in multiple tumor cell lines, with noncancerous cell lines as the baseline control. Most tumor cell lines had higher STING than noncancerous cell lines (the average value of mRNA from multiple cell lines). (i) A detailed bar chart to show the STING mRNA transcript in eight CESC cell lines and 63 noncancerous cell lines. (j) On average, CESC cell lines showed significantly higher STING mRNA than noncancerous cell lines. Each value represents mean ± S.D. We compared multiple groups using one‐way ANOVA with Dunnett’s post hoc test for multiple comparisons. ^∗∗∗^, *p* < 0.001; ^∗∗^, *p* < 0.01; ^∗^, *p* < 0.05.

**Figure:**
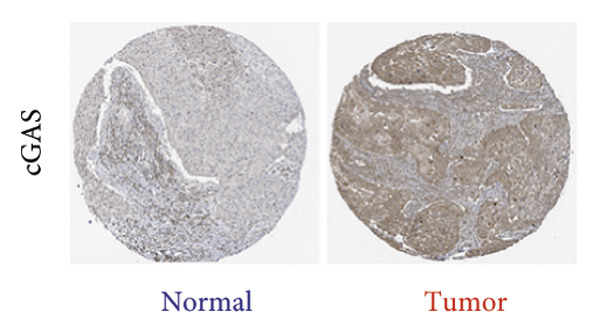
(a)

**Figure:**
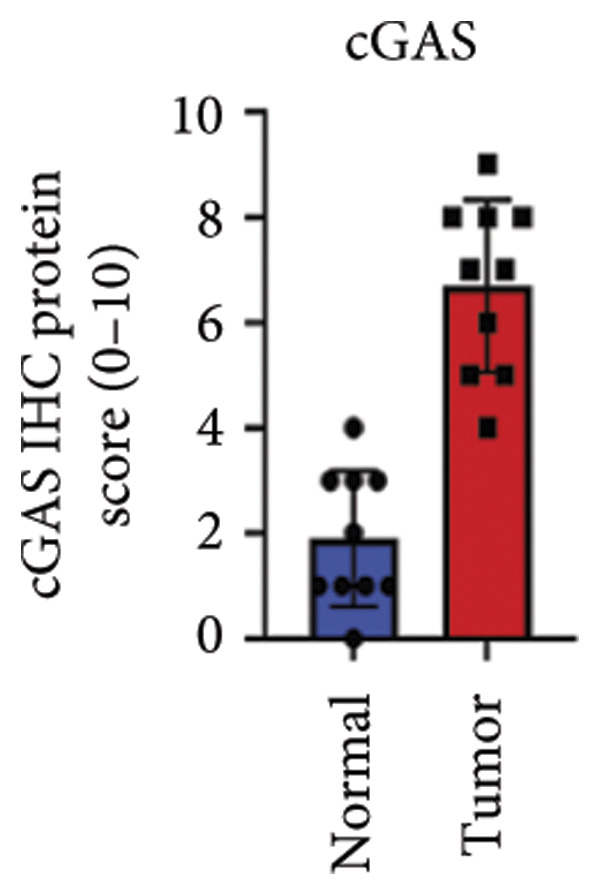
(b)

**Figure:**
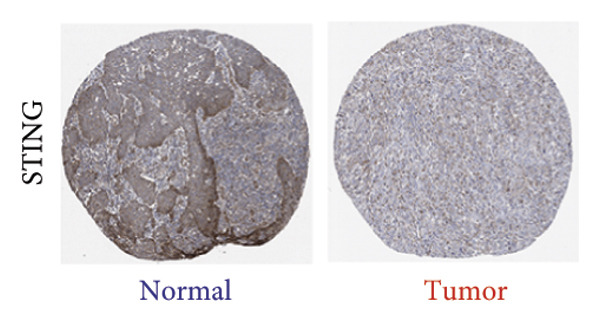
(c)

**Figure:**
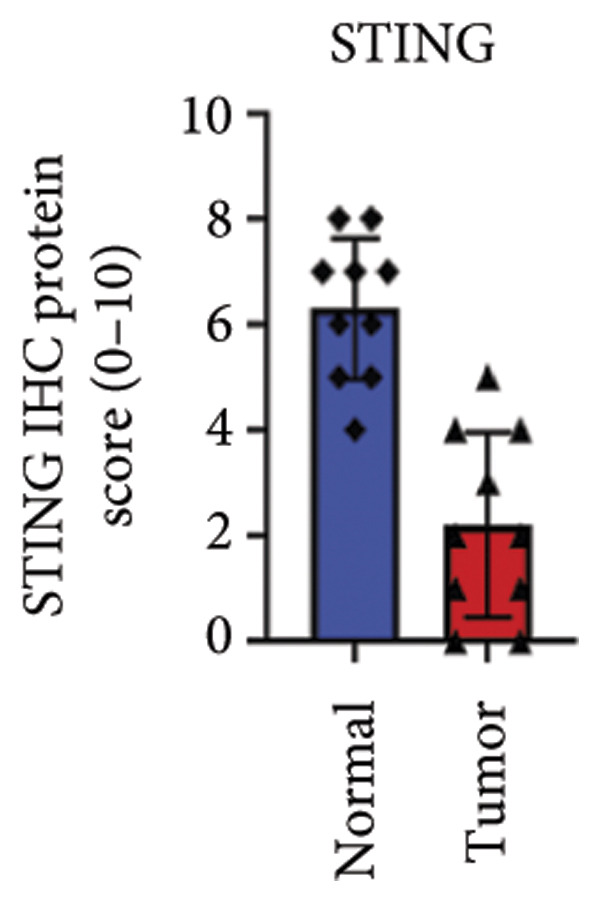
(d)

**Figure:**
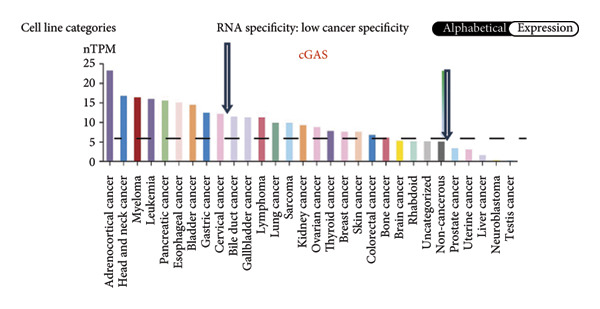
(e)

**Figure:**
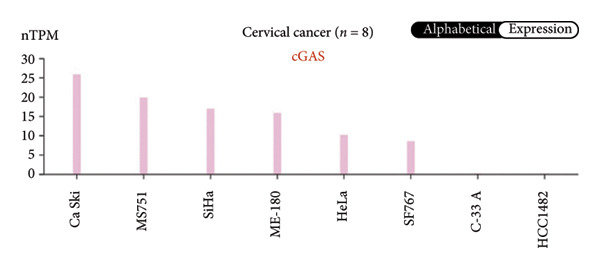
(f)

**Figure:**
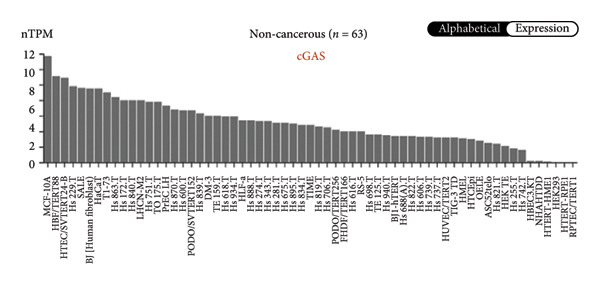
(g)

**Figure:**
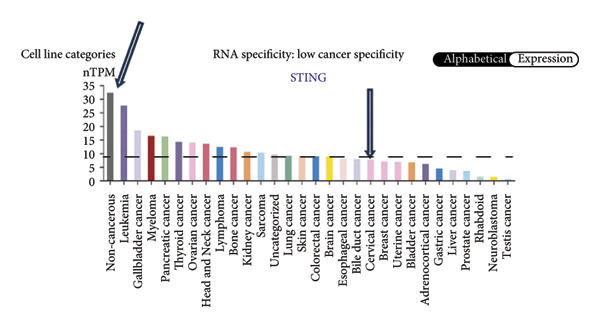
(h)

**Figure:**
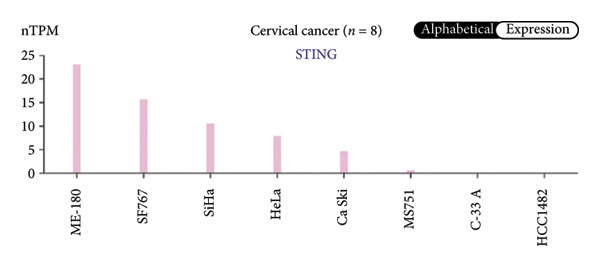
(i)

**Figure:**
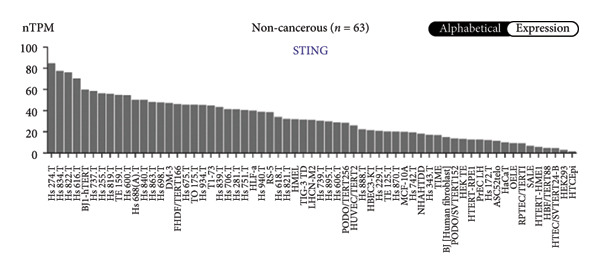
(j)

### 3.4. STING Downstream Pathways Correlate With T Cell Infiltration and Prognosis

It is widely recognized that the activation of the cGAS–STING signaling pathway can stimulate TBK1‐IRF3 and induce Type I IFN signaling. This process promotes the production of chemoattractant (e.g., CCL5) [22] and chemokines (e.g., CXCL9 and CXCL10) [23] that facilitate T cell trafficking and infiltration to the tumor site. In order to verify this, we investigated the correlation between TBK1, IRF3, CCL5, CXCL9, and CXCL10 and the survival of patients in CESC using an online tool, Kaplan–Meier Plotter (https://kmplot.com/analysis/index.php?pservice [19]). Our findings demonstrated that TBK1, IRF3, CCL5, CXCL9, and CXCL10 all had prognostic value in CESC patients. Patients with high TBK1, IRF3, CCL5, CXCL9, and CXCL10 expression exhibited improved OS (Figures 4(a), 4(c)) and RFS (Figures 4(b), 4(d)) compared with those with low TBK1, IRF3, CCL5, CXCL9, and CXCL10 expression in CESC. It is widely recognized that a high STING level can facilitate T cell infiltration and promote tumor eradication [24–26]. To validate this, we conducted an analysis on the relationship between STING level and CD8^+^T and CD4^+^T infiltration using TIMER 2.0 (http://timer.cistrome.org [20]). As illustrated in the two heat maps, our data indicated that the tumor microenvironment with high STING also exhibited a high infiltration of CD8^+^T and CD4^+^T cells (Figures 4(e), 4(f)).

Figure 4STING downstream genes are correlated with the overall survival, relapse‐free survival, and CD4^+^T and CD8^+^T cell infiltration in cervical cancer. (a, b) The overall survival (OS) and relapse‐free survival (RFS) of cervical cancer patients stratified by the expression of TBK1 and IRF3, downstream of STING. Graphs were generated from the Kaplan–Meier survival analysis dataset. (c, d) The overall survival (OS) and relapse‐free survival (RFS) of cervical cancer patients stratified by the expression of CCL5, CXCL9, CXCL10, chemoattractants, and chemokines produced by STING activation. Graphs were generated from the Kaplan–Meier survival analysis dataset. (e, f) The correlation of STING gene and immune infiltration characteristics in a variety of tumor types. Gene expression was standardized, and mean gene values were calculated and logarithmically converted. Comparative expression analysis was conducted using an R‐based methodology with the DeSeq2 tool. (e) The expression of STING is positively relative to the CD8^+^T cell infiltration score as shown in the heat map; data are analyzed in the TIMER 2.0 database. (f) The expression of STING is positively relative to the CD4^+^T cell infiltration score as shown in the heat map; data are analyzed in the TIMER 2.0 database.

**Figure:**
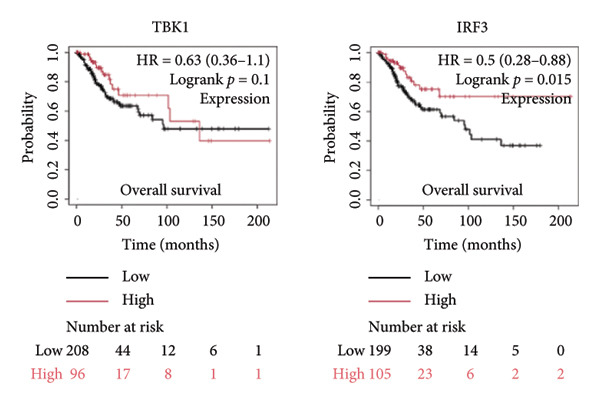
(a)

**Figure:**
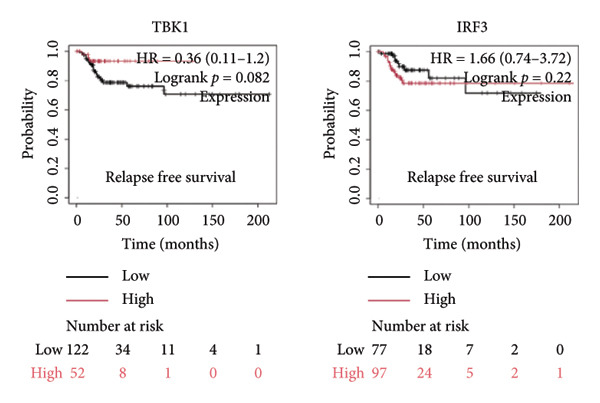
(b)

**Figure:**
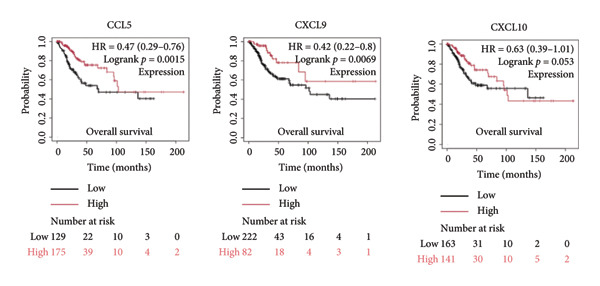
(c)

**Figure:**
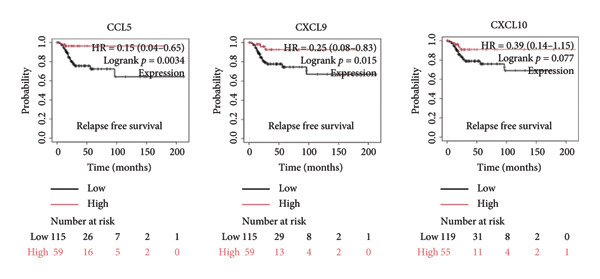
(d)

**Figure:**
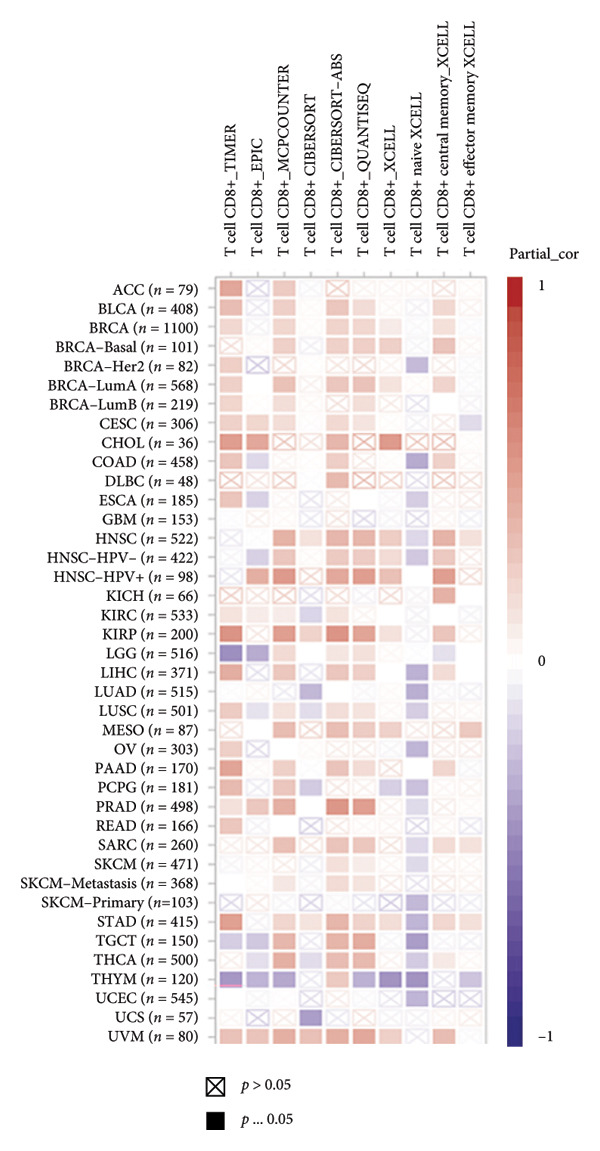
(e)

**Figure:**
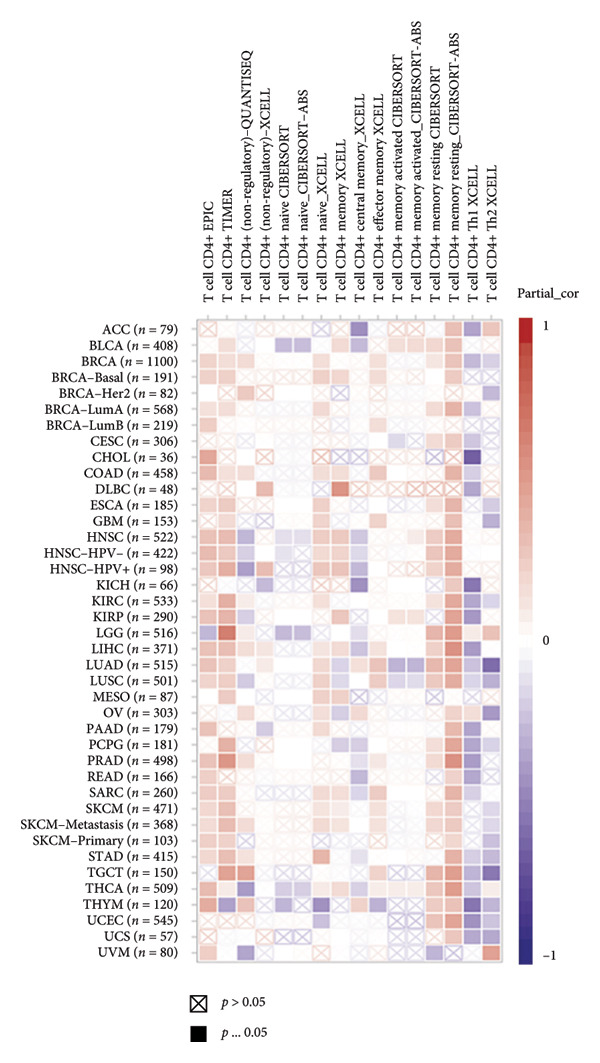
(f)

### 3.5. cGAS and STING DNA Promoter Methylation Show Different Patterns

Activation of the cGAS–STING pathway occurs through the allosteric interaction between cGAS and double‐stranded DNA (dsDNA), which enhances its enzymatic activity and results in the synthesis of 2′3′ cyclic GMP–AMP (cGAMP), which is a second intracellular messenger molecule and acts as a potent agonist of STING (Figure 5(a)) [27, 28]. The downregulation or loss of STING protein expression has previously been reported in a significant proportion of human tumor cell lines, ranging from 50% to 80% for STING [29]. Molecular study of DNA methylation was carried out to investigate the involvement of DNA methylation in the expression of CESC STING and cGAS. We evaluated the prevalence of promoter hypermethylation in cGAS and STING transcripts in humans by analyzing publicly accessible gene methylation databases (https://ualcan.path.uab.edu/cgi-bin/TCGA-methyl-Result.pl [17]) from The Cancer Genome Atlas (TCGA).We observed a major decrease in cGAS promoter methylation (Figures 5(b), 5(c), 5(d), 5(e), 5(f)) and an increase in STING DNA promoter methylation (Figures 5(g), 5(h), 5(i), 5(j), 5(k)), although the latter was not as significant as the former. A negative relationship exists between the methylation of the STING promoter and its mRNA expression, and STING hypermethylation has been observed in numerous cancer types. It is important to mention that the methylation levels were altered in early tumor transformation (Stage CIN1–3/TNM I) patients (*p < *0.01, Figure 5(c)). These statistics suggest the possibility of diagnosing CESC at the early stages by detecting aberrant STING hypermethylation. Subsequently, we evaluated the correlations between clinical characteristics and STING methylation. There were significant changes in cGAS and STING methylation, tumor/normal (*p* < 0.001), tumor TNM stage (*p* < 0.001), tumor grade (*p* < 0.01), and distant metastasis (*p* < 0.001) (Figure 5). These findings collectively imply that hypermethylation of the STING promoter occurs at the early stage of neoplasia and persists as malignant transformation progresses. This suggests a potential role for STING silencing in facilitating immune evasion in CESC.

Figure 5Different methylation patterns of cGAS and STING in cervical squamous tumors. (a) Diagram to illustrate 2′3′cyclic GMP–AMP–STING pathway briefly. (b–f) Graphs to illustrate cGAS DNA promoter methylation with different clinicopathological factors in human using publicly available gene methylation datasets. (b) *cGAS* DNA promoter showed hypomethylated in the CESC tumor. (c) *cGAS* DNA promoter exhibited a gradual decrease in methylation in accordance with the TNM Stages I, II, III, and IV; early‐stage (Stage I/II); and advanced‐stage (Stage III/IV) patients. (d) *cGAS* DNA promoter methylation was lower in tumors with nodal metastasis. (e) *cGAS DNA* methylation showed a decrease with tumor grade. (f) *cGAS* mRNA differed in tumor histology, with endometroid histology type being the highest *cGAS* and mucus the highest. (g) *STING* DNA promoter showed hypermethylated in the CESC tumor. (h) *STING* DNA promoter methylation was higher in tumors with nodal metastasis. (i) *STING* DNA promoter exhibited a gradual increase in methylation according to TNM Stages I, II, III, and IV; early‐stage (Stage I/II); and advanced‐stage (Stage III/IV) patients. (j) *STING* mRNA differed in tumor histology, with adenosquamous histology type being the highest *methylation* and squamous the highest. (k) *STING DNA* methylation showed a decrease with tumor grade.

**Figure:**
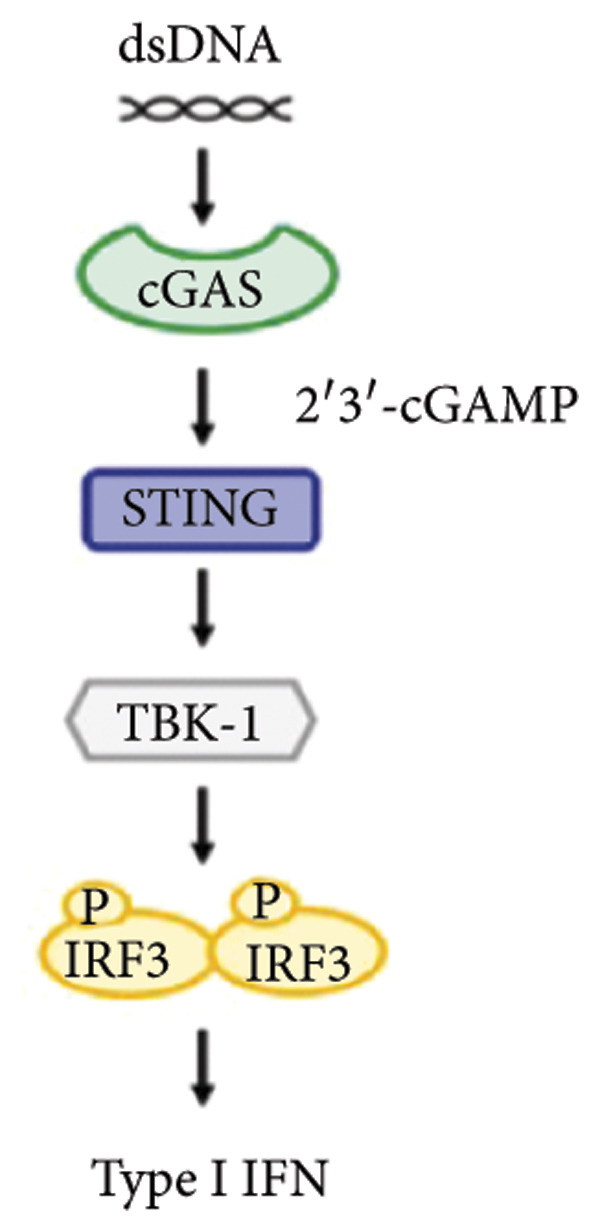
(a)

**Figure:**
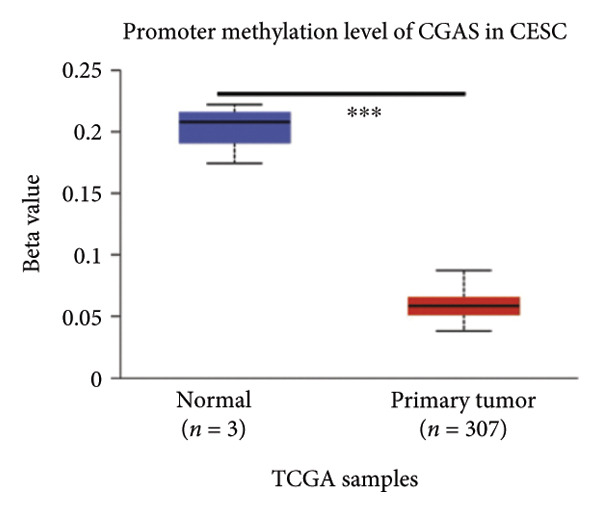
(b)

**Figure:**
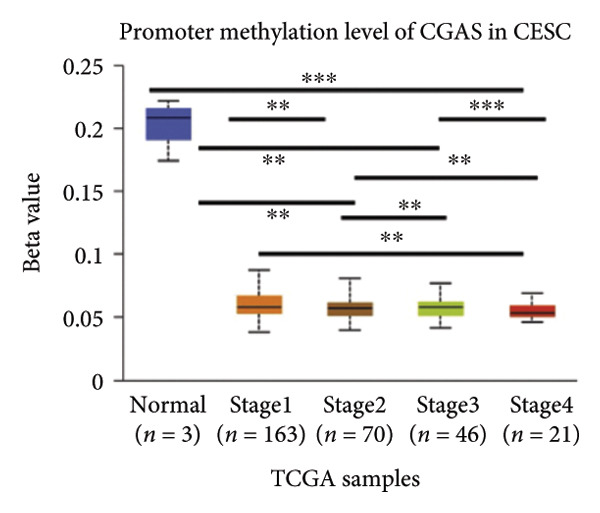
(c)

**Figure:**
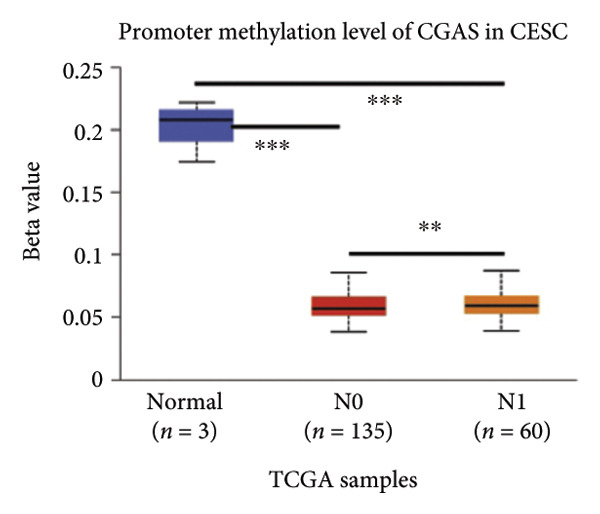
(d)

**Figure:**
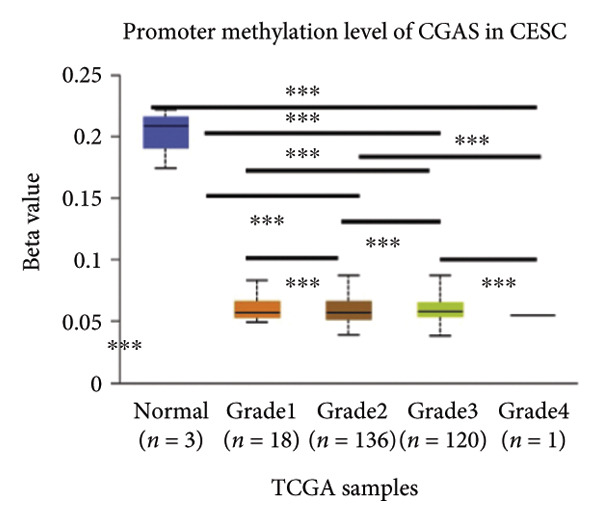
(e)

**Figure:**
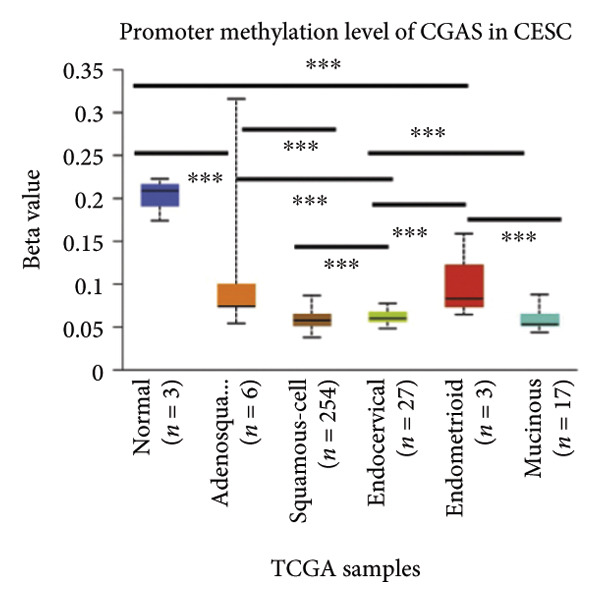
(f)

**Figure:**
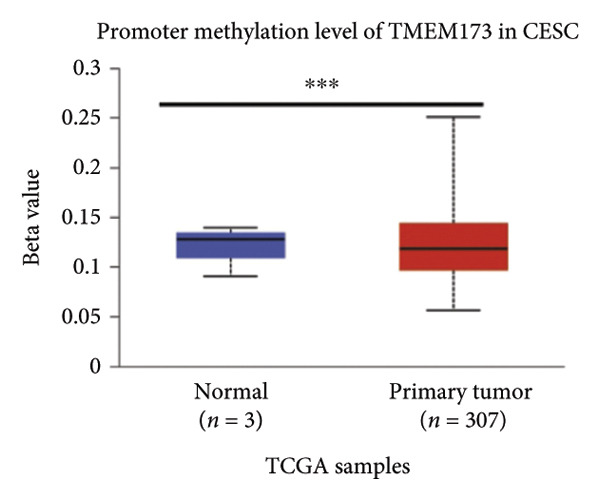
(g)

**Figure:**
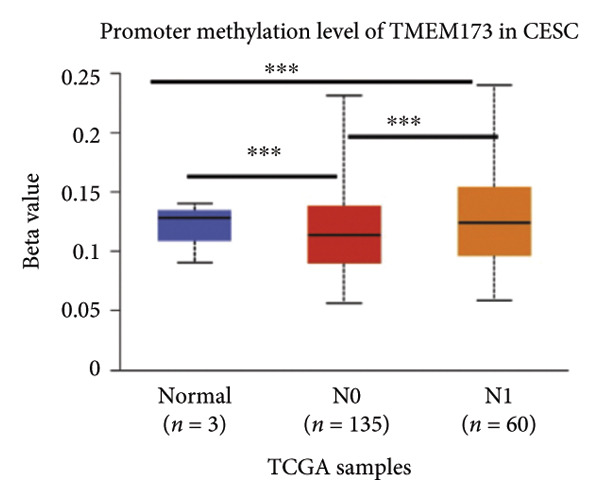
(h)

**Figure:**
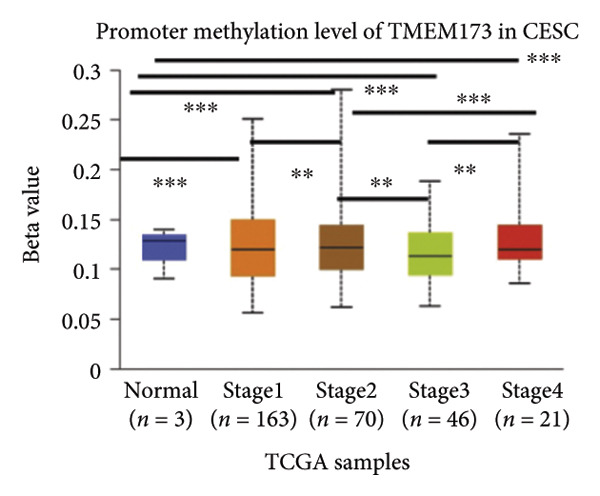
(i)

**Figure:**
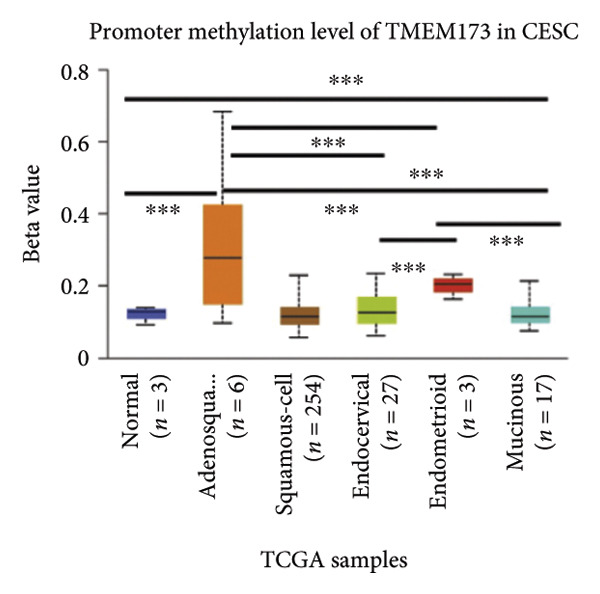
(j)

**Figure:**
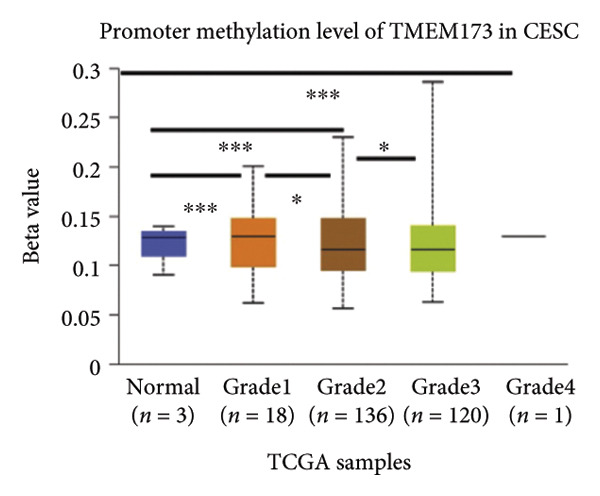
(k)

### 3.6. cGAS and STING IHC Confirms the Same Pattern in CESC Tumor

To verify the findings using public datasets, we retrospectively selected 40 patients with CESC and evaluated cGAS and STING protein expression using IHC in primary tumor samples. A concise summary of the clinical features of the 40 cervical cancer patients and 20 normal controls involved in this study is provided in Table 1. It describes the major clinicopathological parameters that characterize the cohort patients, which include age, FIGO stage, menstruation status, follow‐up time, histology, tumor diameter, and serum tumor markers.

**Table 1:** Patient characteristics.

Characteristics	CESC+ CIN patients Median (range)	Normal control Median (range)
Age (years)	39 (32–68)	40 (29–70)
Menstrual status (premenopausal vs. postmenopausal)	40 (16 vs. 24)	20 (10 vs. 10)
Follow‐up (months)	12 (1–23)	12 (1–23)
FIGO stage		
CIN 1	6	
CIN 2	7	
CIN 3	7	
IA	3	
IB	9	
IIA	5	
IIB	3	
Histology		
Squamous cell carcinoma (number/percentage)	32/40 (80%)	
Serum tumor marker		
CEA (ng/mL)	2.7 (1.1–4.6)	
SCC (ng/mL)	5.6 (1.8–9.9)	

Abbreviations: CEA = carcinoembryonic antigen, CESC = cervical squamous carcinoma, FIGO = International Federation of Gynecology and Obstetrics, SCC = squamous cell carcinoma antigen.

We have previously conducted an analysis of the TCGA dataset and discovered a substantial increase in cGAS mRNA, while STING mRNA showed a significantly decrease by IHC. Tumors were classified according to the TNM Stages CIN1, CIN3, I, and II, and normal cervical tissues were used as a control. Our results indicated a gradual increase in cGAS (Figures 6(a), 6(b)) and a gradual decrease in STING (Figures 6(c), 6(d)). The cGAS and STING staining intensities were quantified from 0 to 10 in CESC Stages CIN1, CIN3, I, and II based on TNM staging; each value represents mean ± S.D (Figures 6(e), 6(f)). Representative IHC images of cGAS staining in normal cervical tissues and cervical squamous tumors of Stages CIN1, CIN3, I, and II according to TNM staging with a resolution of 40× and 200× are shown in Figures 6(a) and 6(b), and representative IHC images of STING staining in normal cervical tissues and cervical squamous tumors of Stages CIN1, CIN3, I, and II according to TNM staging with a resolution of 40× and 200× are shown in Figures 6(c) and 6(d).

Figure 6CESC tumor exhibits high cGAS and low/negative STING by IHC. (a, b) Representative immunohistochemistry images of cGAS staining in normal cervical tissues, and cervical squamous tumors of Stages CIN1, CIN3, I, and II according to TNM staging with a resolution of 40× and 200×. (c, d) Representative immunohistochemistry images of STING staining in normal cervical tissues and cervical squamous tumors of Stages CIN1, CIN3, I, and II according to TNM staging with a resolution of 40× and 200×. (e, f) cGAS and STING IHC staining intensities in A, B, C, D were quantified from 0 to 10 score in CESC Stages CIN1, CIN3, I, and II in accordance with TNM staging. Ten samples each from the normal control group, CIN1, CIN3, and Stages I and II were included for immunohistochemical staining. The results revealed a progressively intensifying staining pattern for cGAS, whereas STING exhibited a gradually diminishing trend. Each value represents mean ± S.D. We compared multiple groups using one‐way ANOVA with Dunnett’s post hoc test for multiple comparisons. ∗∗∗, *p* < 0.001; ∗∗, *p* < 0.01; ∗, *p* < 0.05.

**Figure:**
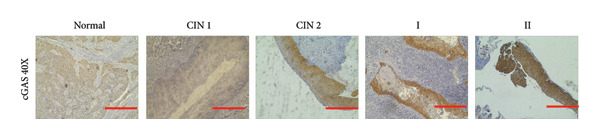
(a)

**Figure:**
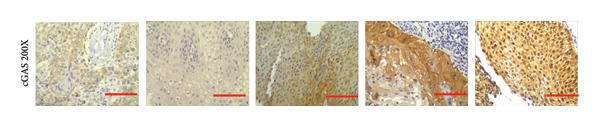
(b)

**Figure:**
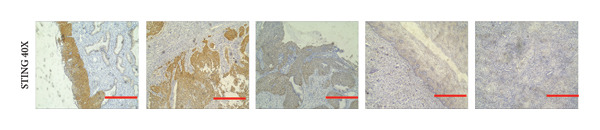
(c)

**Figure:**
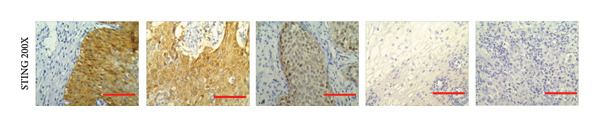
(d)

**Figure:**
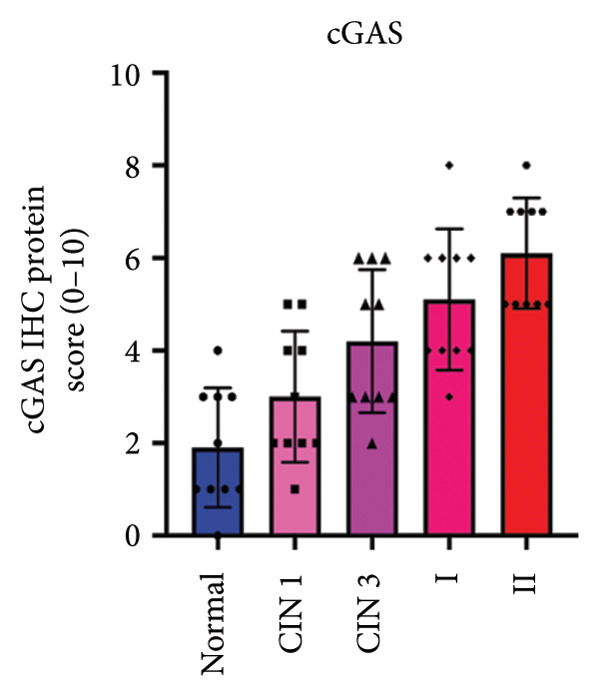
(e)

**Figure:**
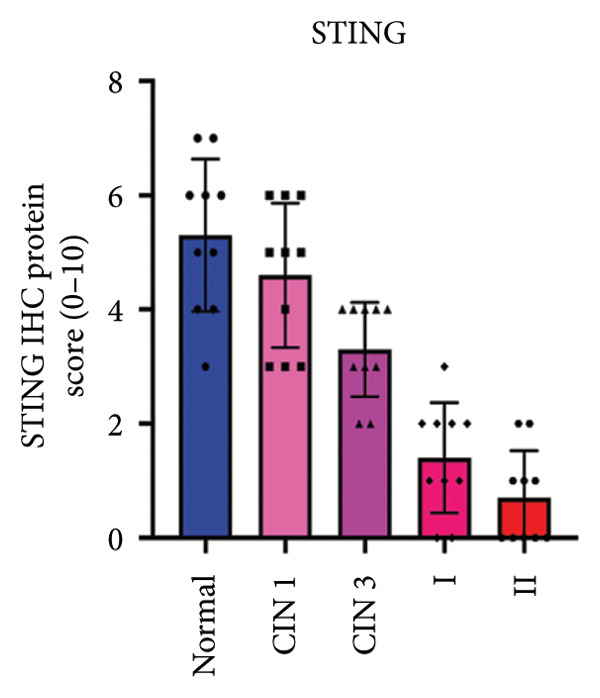
(f)

## 4. Discussion

In this study, we explored the pathway of innate immune stimulator of IFN genes (STING) and the DNA methylation pattern in CESC and revealed significant variations in the main human clinical samples. We found that STING expression is downregulated in cervical tumor cell lines and squamous tumor patient samples, with high tumoral STING expression correlating with T cell infiltration and good RFS and OS. On the contrary, cGAS as an upstream enzyme to activate STING, its expression is upregulated in cervical tumor cell lines and squamous tumor patient samples, with high tumoral cGAS expression correlating with poor RFS and OS. This is a novel discovery in the cGAS–STING innate immune signaling, as cGAS–STING activation is considered to boost IFN production and pro‐inflammatory immune cell infiltration, which helps eradicate tumor cells.

Cervical cancer ranks as the fourth leading cause of tumor‐related deaths in women and is the most widespread form of gynecological cancer globally [2]. Cervical cancer is mostly attributed to chronic infection with the HPV, with Type 16 and 18 viral variants representing around 70% of all cervical malignancies. The overall frequency of HPV in cervical cancer has been reported by several studies to reach up to 99%, suggesting that ongoing infection with HPV plays a crucial role in the development of cervical cancer [3]. However, not all HPV infections result in malignant tumor development. A substantial percentage of HPV infections, approximately 90%, is eliminated within 2 years, indicating that the immune system is generally effective in eradicating the virus. Furthermore, apart from its role in carcinogenesis prevention through the eradication of HPV infection, the immune system also plays a crucial role as a defense against cancer by identifying and eliminating transformed cells in the process of early/developing malignancies.

Deficient STING is reported to be associated with tumor growth and impaired DNA virus sensing function [30]. Previous research has demonstrated that STING functions by dynamically modulating cytokine production. IFNs are multifunctional cytokines that participate in the suppression of viral and tumor progression, as well as the modulation of cellular and immunological responses. Specifically, Type 1 IFNs (IFN‐α and IFN‐β) have been suggested to suppress the growth of cancer cells and trigger cell death processes [31]. CD8^+^ T cell depletion has been reported to impede the control of injected tumors provided by ADU‐S100 [32]. The availability of tumor immunotherapy was restricted by the insufficient infiltration of T lymphocytes in tumors and the escape of tumor cells from host immunosurveillance.

STING is an innate immune sensor of cytoplasmic second messenger cGAMP. The activation of cytosolic DNA–STING pathway in tumor microenvironments usually leads to more robust adaptive immune responses to tumors. Numerous malignancies, such as melanoma, colorectal cancer, and lung cancer with KRAS/LKB1 mutations, exhibit suppression of STING signaling [30]. STING suppression is achieved through a variety of mechanisms, such as DNA methylation of the promoters of STING (STING1) [33] and loss‐of‐function gene mutations that encode these proteins [34]. The immunosuppressive microenvironment of cervical cancer significantly hampers the efficacy of immunotherapy. We discovered a unique strategy to boost host innate immune functions and restore cancer immunogenicity by increasing STING expression.

The protein expression of cGAS is controlled by post‐translational modifications. The primary activation mechanism of cGAS requires its contact with dsDNA. To maintain a strong and sensitive response to foreign DNA while avoiding reactivity to self‐DNA, the cGAS pathway must be controlled by complementary processes [35]. Previous research has shown that the enzymatic activity of cGAS is regulated by post‐translational changes; however, our understanding of cGAS regulation is still in its early stages. The kinase Akt26 phosphorylates human cGAS at Ser305 (Ser291 in rodent cGAS) [36]. This phosphorylation inhibits the activity of cGAS, which suggests that there may be possible intercommunication between the cGAS pathway and other Akt‐regulating pathways [36].

Despite our important findings presented here, there are some limitations in this study. The cGAS and STING DNA methylation is not confirmed in clinic samples, with detailed cGAS and STING DNA methylation sites not investigated. Further study to explain why cGAS and STING have different expressions and methylation patterns are necessary. In addition, more molecular mechanisms were necessary to clarify how cGAS and STING might play different roles in facilitating or inhibiting tumorigenesis and thus may translate into therapeutic treatments that targeted cGAS–STING signals applied to cervical squamous tumors. Of note, our results indicate a potential for cGAS inhibitor development for CESC tumors.

## Ethics Statement

The present study has obtained ethical permission and consent from the Ethics Committee of Suqian Affiliated Hospital of Xuzhou Medical University (Approval Number 2023‐S075). Each participant in this study was given detailed information about the study’s purpose and methodology and obtained consent following the hospital’s ethical standards. The processes mentioned above were conducted in accordance with the relevant protocol and the Declaration of Helsinki.

## Disclosure

All authors have read and approved the final manuscript. The funders/sponsors had no role in the design of the study; in the collection, analyses, or interpretation of data; in the writing of the manuscript; or in the decision to publish the results.

## Conflicts of Interest

The authors declare no conflicts of interest.

## Author Contributions

Shuling Liu and Ruimin Wang conceived and designed this study and contributed to data collection and interpretation. Rui Wang performed statistical analysis and wrote the draft. Quanquan Guo and Xiaoyuan Lu interpreted data and revised the manuscript. All authors assume full responsibility for the integrity of the data and the accuracy of the analytical findings, with complete access to the study data granted to all contributors. Shuling Liu and Ruimin Wang are co‐first authors.

## Funding

This research received financial assistance from the Natural Science Foundation of Suqian Science and Technology Bureau through Grant No. K202410 and Scientific and Technological Association of Jiangsu through Grant No. JSTJ‐2022‐004. Financial support for Rui Wang was provided by the China Scholarship Council (Grant No. 20220692039).

## Data Availability

We declare that all data necessary to replicate the findings reported in this manuscript are available from the Human Protein Atlas (https://www.proteinatlas.org), UALCAN database (http://ualcan.path.uab.edu/analysis-prot.html), and Kaplan–Meier Plotter (https://kmplot.com/analysis/index.php?pservice). Patients’ sample IHC will be provided on reasonable request.
